# Anwendung stark wirksamer retardierter Opioide in der Pädiatrie: Fallstricke und Lösungen für Morphin und Hydromorphon

**DOI:** 10.1007/s00482-023-00775-w

**Published:** 2024-01-03

**Authors:** Franziska Zimbelmann, Sarah Flaute, Melanie Deipenbrock, Elvira Ahlke, Georg Hempel, Margit Baumann-Köhler

**Affiliations:** 1 Spezialisierte Ambulante Palliativversorgung für Kinder und Jugendliche (SAPV), Pädiatrische Hämatologie und Onkologie, Zentrum für Kinder- und Jugendmedizin, Universitätsklinikum Münster (UKM), Münster, Deutschland; 2 Institut für Pharmazeutische und Medizinische Chemie, Klinische Pharmazie, Universität Münster, Corrensstraße 48, 48149 Münster, Deutschland; 3 Rezeptur/Sterilherstellung, Eberwein & Plassmann OHG, Hohenzollern Apotheke, Münster, Deutschland; 4 Pädiatrische Hämatologie und Onkologie, Zentrum für Kinder- und Jugendmedizin, Universitätsklinikum Münster (UKM), Münster, Deutschland

**Keywords:** Morphin, Hydromorphon, Dosisteilung, HPLC-Analytik, Gastrointestinale Sonden, Morphine, Hydromorphone, Dose partition, HPLC-analysis, Gastrointestinal tubes

## Abstract

**Hintergrund:**

In der Pädiatrie bedarf es zur adäquaten Therapie mit stark wirksamen Opioiden für viele Patienten der Gabe von retardierten Präparaten. Dabei stellen die Dosierbarkeit und Sondierbarkeit die Versorger vor eine große Hürde, insbesondere nachdem die Firma Mundipharma GmbH die Produktion und den Vertrieb des für diese Zwecke in der Pädiatrie bewährten Präparats MST®-Retardgranulat 2019 eingestellt hat. Ziel der Untersuchungen war es, ein sicheres Dosisteilungsverfahren von auf dem Markt erhältlichen und sondierbaren retardierten Opioidpräparaten zu erarbeiten, insbesondere für die Anwendung in der Pädiatrie, welche einen Niedrigdosisbereich benötigt.

**Methodik:**

Aus je einem ausgewählten retardierten Morphin- und Hydromorphonpräparat wurden durch Öffnen der Kapseln und Abwiegen der retardierten Pellets Abfüllungen mit niedrigen Dosierungen hergestellt. Anschließend wurde zur Beurteilung der Dosisteilung der Arzneistoffgehalt jeder Abfüllung mittels Hochleistungsflüssigkeitschromatographie(HPLC)-Analytik bestimmt. Weiterhin wurde die Sondierbarkeit der Pellets über gastrointestinale Sonden (Charrière Ch 8–Ch 10) in einem *Ex-vivo*-Versuch untersucht.

**Ergebnisse:**

Die Untersuchungen zeigten ein praktikables Verfahren zur Rezepturherstellung niedrig dosierter Abfüllungen von retardiertem Morphinsulfat bzw. Hydromorphonhydrochlorid (Hydromorphon-HCl). Die Abfüllungen entsprechen der vom Europäischen Arzneibuch (EuAB) vorgeschriebenen Prüfung auf Gleichförmigkeit des Gehalts einzeldosierter Arzneiformen. Weiterhin passierten die Pellets mittels Spritzenapplikationstechnik im *Ex-vivo*-Versuch Sonden mit Ch 8 (Morphinsulfat) und Ch 10 (Hydromorphon-HCl).

**Diskussion:**

Das Verfahren kann als sicher angesehen werden und bietet somit eine Möglichkeit, in der Pädiatrie retardierte stark wirksame Opioide zur oralen Applikation und Applikation per Sonde im „off label use“ einzusetzen.

## Abstract (en)

**Background:**

In pediatrics, adequate treatment with potent opioids requires the administration of sustained-release preparations for many patients; however, the dosing and administration of sustained-release morphine and hydromorphone preparations via gastrointestinal tubes confronts providers with a major hurdle, especially as the company Mundipharma GmbH has discontinued the production and distribution of the preparation MST retard granules in 2019, which has been proven for these purposes in pediatrics. The aim of this study was to establish a production technique for available sustained-release opioid preparations, which are particularly suitable for use in the low-dose range required in pediatrics and which can also be administered via gastrointestinal tubes.

**Method:**

Low-dose preparations were produced by opening of morphine and hydromorphone capsules and weighing of the sustained-release pellets. To evaluate the partition, an analysis of the drug content via high performance liquid chromatography (HPLC) was conducted. Moreover, the administration via gastrointestinal tubes (charrière, Ch 8–Ch 10) was examined by an ex vivo experiment.

**Results:**

The examination showed a practicable method to produce low dosages of sustained-release morphine and hydromorphone. The preparations are in accordance with the test for content uniformity of the European Pharmacopoeia (Ph. Eur.). Furthermore, the pellets were administered to gastrointestinal tubes Ch 8 (morphine) and Ch 10 (hydromorphone) by a syringe application technique and passed the tubes completely.

**Conclusion:**

The production technique can be considered as safe and enables the off-label oral application or application via gastrointestinal tubes of sustained-release opioids in pediatrics.

**Graphic abstract:**

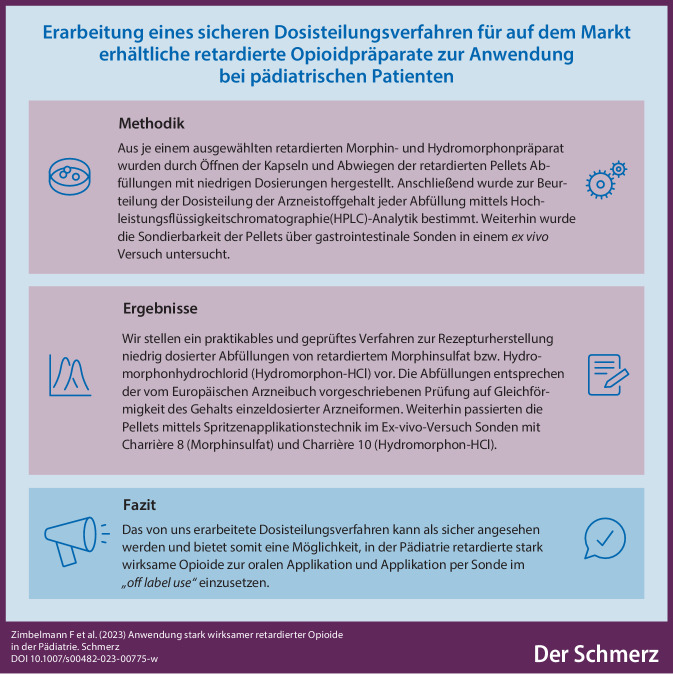

## Hintergrund und Fragestellung

Die Behandlung von Schmerz und Dyspnoe mit stark wirksamen Opioiden stellt in der Pädiatrie in Bezug auf Dosierung, Sondierbarkeit und Sicherheit eine besondere Herausforderung dar. 2019 hat die Firma Mundipharma GmbH ihr Präparat MST®-Retardgranulat vom Markt genommen, das als einziges retardiertes Präparat eine Dosierung und Verabreichung auch über gastrointestinale Sonden in einem Dosisbereich < 10 mg ermöglichte. In diesem Beitrag wird die Untersuchung zu auf dem Markt erhältlichen retardierten Morphin‑/Hydromorphonpräparaten in Bezug auf Teilbar- und Sondierbarkeit vorgestellt.

In der Versorgung von Kindern und Jugendlichen mit onkologischen Erkrankungen besteht bei den therapieverantwortlichen Kinderonkologen bzw. -palliativmedizinern in Deutschland eine breite Erfahrung in der Anwendung von Morphin und Hydromorphon. Darüber hinaus haben aber ebenso Kinder und Jugendliche mit schweren chronischen und meist lebensbegrenzenden Erkrankungen anderer Fachgebiete der Pädiatrie, wie Neurologie oder Stoffwechselmedizin, einen Bedarf an und einen Anspruch auf eine adäquate Supportiv- und Schmerztherapie, wenn nötig auch mit stark wirksamen Opioiden [15]. Viele der betroffenen Kinder leiden unter ausgeprägten Schluckstörungen und werden daher über gastrointestinale Sonden mit Nahrung, Flüssigkeit und Medikamenten versorgt. Im Säuglingsalter werden dazu meist zunächst nasogastral liegende Sonden und für die längerfristige Versorgung eine perkutane endoskopische Gastrostomie (PEG) und/oder perkutane endoskopische Jejunostomie (PEJ) angelegt. Vor der Verordnung und Gabe eines Medikaments und besonders retardierter Präparate muss sichergestellt sein, dass die Formulierung sondengängig für die vorliegende Sondenform und -größe ist, da ansonsten eine Okklusion mit eventuell weitreichenden Konsequenzen für die Versorgungssituation des Kindes eintreten kann.

Wie bei Erwachsenen ist bei Kindern ein individualisiertes Schmerz‑/Dyspnoetherapiekonzept mit einer Basis- und Bedarfsmedikation zu erstellen, wenn nötig und möglich unter Einsatz retardierter Präparate. Eine grundsätzliche Limitierung in der Auswahl des Arzneimittels besteht dabei in der Pädiatrie fast regelhaft, da die entsprechenden Arzneimittel meist außerhalb der Zulassung („off label“) zur Verfügung stehen und die Studienlage zu ihrer Anwendung sehr eingeschränkt ist [9].

Morphin ist in den Indikationen Schmerzen und Dyspnoe in allen Altersgruppen der Pädiatrie das am häufigsten eingesetzte Opioid, für das retardierte, orale Präparate erhältlich sind [2, 14]. Hydromorphon wird je nach Altersgruppe und Grunderkrankung vermehrt primär oder als Kandidat im Rahmen einer Opioidrotation eingesetzt [8]. Fentanyl und Buprenorphin, in der Form transdermaler Pflaster, finden daneben, ab einem oralen Tagesmorphinäquivalent von 30 mg für Fentanyl und 10 mg für Buprenorphin, seltener ebenfalls eine Anwendung in der Pädiatrie [5, 12].

Besondere Beachtung und Sorgfalt des Verordnenden erfordert darüber hinaus, dass in der Pädiatrie *per se* ein erhöhtes Risiko für inadäquate Dosierungen mit der Folge unzureichender Wirkung oder lebensbedrohlicher Überdosierungen und Nebenwirkungen besteht. Dies gilt insbesondere für Medikamente mit einer geringen therapeutischen Breite wie stark wirksame Opioide [10].

Bis 2019 war in Deutschland, der Schweiz und Österreich ein retardiertes Morphinpräparat (Mundipharma GmbH) erhältlich, das eine feine Dosierung in 0,1 mg-Schritten und eine Gabe über gastrointestinale Sonden auch mit kleinen Durchmessern wie 8 Charrière (Ch) ermöglichte. Mit der Einstellung der Produktion und des Vertriebs durch die Firma Mundipharma standen somit die pädiatrischen Patienten und Versorger vor dem Dilemma, auf kein erprobtes retardiertes Präparat zurückgreifen zu können, mit dem eine adäquate Dosierbarkeit im Bereich unter 10 mg und wenn notwendig auch eine Sondierung umgesetzt werden konnte.

Inhalt dieser Untersuchung ist es, eine Auswahl von auf dem Markt verfügbaren Retardpräparaten für Morphin und Hydromorphon auf ihre sichere Teilbarkeit und somit Dosierbarkeit, aber auch auf ihre Sondengängigkeit hin zu überprüfen.

## Methodik

In der Untersuchung wurden aus mit retardiertem Morphin und Hydromorphon gefüllten Kapseln Abfüllungen mit geringeren Dosierungen hergestellt und anschließend der Gehalt der Abfüllungen bestimmt. Weiterhin wurden die verwendeten Pellets auf ihre Sondierbarkeit geprüft. Die verwendeten Fertigarzneimittel (Capros®-10 mg-Hartkapseln retardiert [Wirkstoff: Morphinsulfat] und Hydromorphon-HCl-beta-2 mg-Retardkapseln [Wirkstoff: Hydromorphon-HCl]) wurden aufgrund von Beschreibungen der Sondierbarkeit im pädiatrischen Ch-Bereich ausgewählt. Bei beiden Produkten handelt es sich um Kapseln, in denen die retardierte Freisetzung der Wirksubstanzen über eine Pelletformulierung realisiert ist. Die Pellets sind leicht aus der Kapsel zu entnehmen und passieren die typischen pädiatrischen Ch-Bereiche. Die anderen Fertigarzneimittel haben diese Voraussetzungen z. T. nicht erfüllt.

Die Fachinformation von Capros® beschreibt eine mögliche Sondengängigkeit für Sonden ≥ Ch 16 unter Nachspülung von Wasser [4]. Eine Anfrage bei der Arzneimittelinformation Palliativmedizin des Kompetenzzentrums Palliativpharmazie, Ludwig-Maximilians-Universität (LMU) Klinikum München, ergab zudem eine mögliche Sondierbarkeit im „off label use“ unter Zuhilfenahme eines viskosen Hydroxyethylcellulose(HEC)-Gels 4 % bis zu Ch 8 und eines viskosen HEC-Gels 4 % nach 1:2-Verdünnung bis Ch 6,5.

Die Anfrage ergab weiterhin für Hydromorphon je nach Hersteller unterschiedliche Angaben zur Sondierbarkeit (jeweils „off label use“). Für Hydromorphonretardkapseln zweier Hersteller wurde eine Sondengängigkeit bei gastralen Sonden bis Ch 14 bzw. 12 dokumentiert. Für Hydromorphon-HCl beta® wurde eine Applikation über eine Sonde mit Ch 9 untersucht, wobei diese aufgrund der höheren Viskosität gegenüber Wasser mittels Gel, Lactulose oder einer Wasser-Öl-Emulsion (1:1) erfolgen soll [1]. Aufgrund dieser Angaben wurden alle Versuche mit den genannten Handelspräparaten durchgeführt.

### Dosisteilung von Fertigarzneimitteln durch Abwiegeverfahren

#### Herstellverfahren zur Dosisteilung

Die entsprechenden Retardkapseln wurden geöffnet und die einzelnen Pellets so eingewogen, dass die Gesamtmenge an Morphinsulfat entweder 5 oder 7,5 mg und die Gesamtmenge an Hydromorphon-HCl 0,5, 1 oder 1,5 mg betrug. Die ausgewählten Gesamtmengen wurden durch die Mindesteinwaage für die Analysenwaage (mind. 10 mg Fertigarzneimittel) limitiert. Somit wäre eine Einwaage von < 5 mg Morphinsulfat des o. g. Fertigarzneimittels durch das Verfahren der Einwaage nicht darstellbar. Anschließend wurden die Einzeldosen quantifiziert, um die Gleichförmigkeit des Gehalts zu beurteilen.

Um eine statistische Auswertung zu ermöglichen, wurden die Versuche zur Teilbarkeit in 3 unabhängigen Herstellungen (*n* = 6) mit jeweils 10 Abfüllungen pro Stärke durchgeführt.

Es wurden je 20 Retardkapseln geöffnet, die retardierten Pellets in einem Becherglas vereinigt und vorsichtig durchmischt. Die Masse des gesamten Kapselinhalts (= retardierte Pellets) der 20 entleerten Kapseln wurde mittels Analysenwaage bestimmt.

Durch Ermittlung des Gesamtgewichts der Pellets konnte die notwendige Einwaagemenge von Capros® 10 mg für 5 und 7,5 mg Morphinsulfat und von Hydromorphon-HCl beta 2 mg für 0,5, 1,0 und 1,5 mg berechnet werden.

Die entsprechende Pelletmenge wurde direkt in das Endgefäß, eine Schraubdeckeldose mit Originalitätsverschluss, eingewogen, verschlossen und etikettiert. Die überschüssigen Pellets wurde gemäß den rechtlichen Vorgaben vernichtet und entsprechend dokumentiert.

Die Berechnung zur Ermittlung der notwendigen Einwaagemenge an Pellets nach Öffnung von 20 Kapseln Capros® 10 mg ergibt sich exemplarisch wie folgt:

$$\mathrm{m}_{\text{Pellets gesamt}}\cong 200\,\text{mg Morphinsulfat }\cong 20\,\text{Kapseln Capros }10\mathrm{\,mg}$$

$$5\,\text{mg Morphinsulfat }\cong m_{\text{Pellets gesamt}}/200\text{\,mg * }5\mathrm{\,mg}$$

$$7{,}5\,\text{mg Morphinsulfat }\cong m_{\text{Pellets gesamt}}/200\text{\,mg * }7{,}5\mathrm{\,mg}$$

Aufgrund der geringen Wirkstoffmenge pro Einzeldosis wurde die Mindestansatzmenge von je 20 Kapseln pro Herstellung gewählt, um mögliche Schwankungen im Wirkstoffgehalt der einzelnen Pellets zu minimieren. Dies entspricht der gültigen Empfehlung, bei der Herstellung von Kapseln aus Fertigarzneimitteln eine Mindestzahl von 10 Tabletten zu verwenden [13].

### Qualitätskontrolle der Dosisteilung mittels HPLC-Analytik

In einem zweiten Schritt wurden die so hergestellten Einzeldosen der Hochleistungsflüssigkeitschromatographie(HPLC)-Analytik zugeführt. Mit diesem Verfahren wurde der Fragestellung nachgegangen, ob nach dem oben beschriebenen Teilungsverfahren der Präparate eine gleichmäßige Verteilung der Wirkstoffmengen in den Einzeldosen vorlag.

**Abb. 1:**
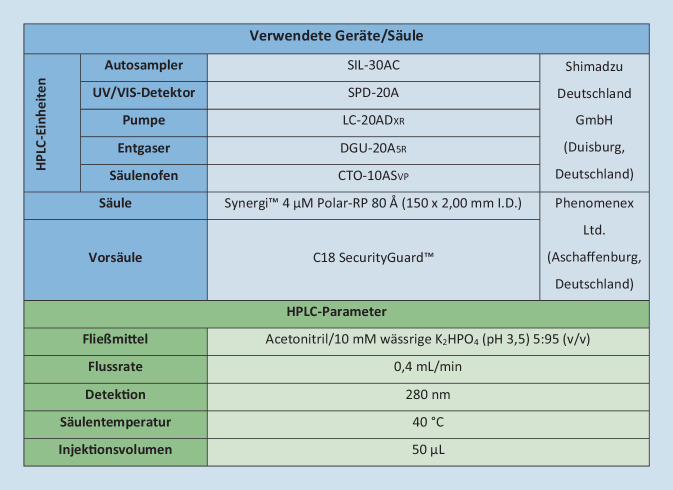
HPLC-Geräte und -Parameter für die isokratische Methode zur Quantifizierung der Opioide

Beide Arzneistoffe lassen sich über dieselbe isokratische HPLC-Methode mit Ultraviolett(UV)/Visible(VIS)-Detektor quantifizieren. Als Grundlage für die hier beschriebene Methode diente die Publikation von Jafari-Nodoushan et al. [6]. Abb. 1 listet die verwendeten Geräte, die Säule und die Parameter zur Quantifizierung der Opioide auf.

Zur Bestimmung der Richtigkeit und Präzision der Methode sind nach den Vorgaben der Food and Drug Administration (FDA) für beide Arzneistoffe Qualitätskontrollproben (QC-Proben) hergestellt und vermessen worden. Für Morphin in jeweils 5‑facher Ausführung MQC-Proben („medium quality control sample“) mit einer Konzentration von 50 mg/l und HQC-Proben („high quality control sample“) mit einer Konzentration von 75 mg/l. Für Hydromorphon analog in jeweils 5‑facher Ausführung LQC-Proben („low quality control sample“) und MQC-Proben, bei denen die Konzentrationen bei 25 mg/l bzw. 50 mg/l lagen. Als Lösungsmittel wurde ein Gemisch aus bidestilliertem Wasser und Methanol in einem Verhältnis von 90:10 (v/v) verwendet.

Die Kalibriergeraden setzten sich aus jeweils 5 Kalibrierpunkten zusammen, bei denen die Analytkonzentration bei 20, 40, 60, 80 und 100 mg/l lag. Auch hier diente als Lösungsmittel das Methanol-Wasser-Gemisch.

Zur Auswertung wurde die Software Labsolutions™ verwendet. Der durchschnittliche Wert für die Richtigkeit nach Vermessung der 5 MQC-Proben von Morphin lag bei 101,5 % (Präzision: 1,4 %). Die Vermessung der 5 HQC-Proben von Morphin ergab eine durchschnittliche Richtigkeit von 99,8 % (Präzision: 6,7 %). Für Hydromorphon ließen sich nach Vermessung der LQC- bzw. MQC-Proben durchschnittliche Werte für die Richtigkeit von 99,4 % (Präzision: 6,7 %) und 99,7 % (Präzision: 1,8 %) ermitteln. Damit erfüllen die Werte für Richtigkeit und Präzision die Anforderungen der FDA [11].

**Abb. 2:**
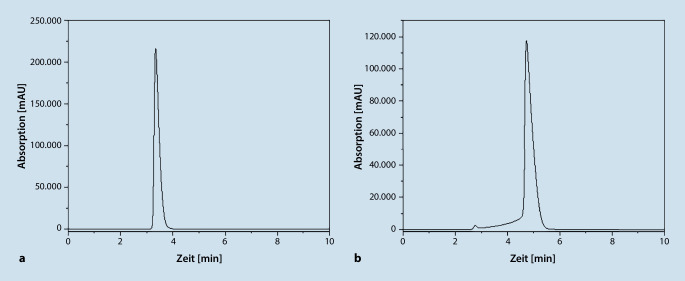
Chromatogramm von Morphin (**a** Retentionszeit: 3,3 min) und von Hydromorphon (**b** Retentionszeit: 4,7 min)

In Abb. 2 sind die Chromatogramme für Morphin und Hydromorphon aufgeführt.

Mit dieser validierten Methode sind die hergestellten Einzeldosen auf ihren Gehalt hin überprüft worden.

#### Qualitätssicherung von patientenindividuell dosierten Abfüllungen

Die einzeldosierten Morphinpellets wurden in 100 ml des Lösungsmittelgemischs aus Wasser und Methanol (90:10 [v/v]), die Hydromorphonpellets jeweils in 20 ml Lösung aufgenommen. Es folgte für alle Proben eine etwa 30-minütige Behandlung im Ultraschallbad und ein anschließendes Erhitzen der Probenlösung auf etwa 70 °C mithilfe eines Magnetheizrührers. Die meisten Morphinpellets zerfielen während der mechanischen Einwirkung im Ultraschallbad, die Hydromorphonpellets hingegen nicht. Eine Trübung der Probenlösung setzte spätestens nach dem Erhitzen der Lösung ein, was die vollständige Lösung des Arzneistoffs aus den Pellets anzeigte.

Die anschließende Analyse mittels HPLC erfolgte in 3‑facher Ausführung pro Probe, sodass aus den ermittelten Arzneistoffkonzentrationen der Mittelwert gebildet und dieser Wert zur weiteren Auswertung verwendet wurde.

### Prüfung auf Sondierbarkeit

Da eine orale Gabe der Opioide nicht für alle pädiatrischen Patienten realisierbar ist, wurde zusätzlich die Applizierbarkeit der verwendeten Präparate über verschiedene gastrointestinale Sonden *ex vivo* untersucht. In der klinischen Praxis werden, entsprechend den Körpermaßen der Kinder- bzw. ihrer individuellen anatomischen und erkrankungsspezifischen Besonderheiten, meist Sonden mit einem Außendurchmesser von 8 bis 12 Ch verwendet und sowohl gastral als auch jejunal platziert. Die untersuchten Sonden bilden ein Teilspektrum der auf dem Markt verfügbaren und häufig eingesetzten Produkte verschiedener Hersteller ab, ohne Anspruch auf Vollständigkeit (Tab. 1). Die Anfrage bei der Arzneimittelinformation Palliativmedizin des Kompetenzzentrums Palliativpharmazie, LMU Klinikum München, beschrieb folgendes Vorgehen bei der Sondierbarkeit von Capros®:

**Tab. 1:** Übersicht der für die Testung verwendeten Sonden. (PUR = Polyurethan)

Sonde	Sondentyp	Material	Ø (außen)	Ø (innen)	Länge	Ø (Konnektor innen)	Ø (Auslassöffnung)
Flocare® Pursoft Tube (Nutricia)	Transnasale Magensonde	PUR	Ch 8	1,7 mm	60 cm	2,9 mm	2,55 × 1,4 mm
Flocare® Nutrisoft Tube (Nutricia)		Silikon	Ch 8	1,8 mm	60 cm	2,8 mm	1,7 mm
Flocare® Pursoft Tube (Nutricia)		PUR	Ch 10	2,1 mm	90 cm	3,0 mm	3,05 × 1,9 mm
Flocare® Nutrisoft Tube (Nutricia)		Silikon	Ch 10	2,3 mm	125 cm	2,9 mm	2,1 mm
Nutrisafe2® PUR (Vygon)		PUR	Ch 8	1,5 mm	75 cm	2,2 mm	2,5 × 1,1 mm
Nutrisafe2® PUR (Vygon)		PUR	Ch 10	2,15 mm	75 cm	2,2 mm	3,3 × 1,5 mm
Freka FKJ (Fresenius)	Perkutane Jejunalsonde	PUR	Ch 9	1,9 mm	75 cm	2,9 mm	1,5 mm

Zuerst wird der Stempel aus der Spritze herausgezogen und diese an der Spritzenöffnung mittels Kombistopper verschlossen. Die Spritze wird in ein Becherglas gestellt und von oben mit 10 ml HEC-Gel befüllt. Die Capros®-Kapsel wird geöffnet, der gesamte Kapselinhalt in die Spritze gegeben und diese bis auf ca. 21 ml mit HEC-Gel aufgefüllt. Nach dem Aufsetzen des Stempels wird die Spritze gedreht und nach ca. 5 s, sobald das Gel etwas abgesunken ist, der Kombistopper entfernt und die Luft aus der Spritze herausgedrückt. Eine Homogenisierung der Suspension wird durch Aufsetzen des Kombistoppers und wiederholtes Herausziehen des Stempels entgegen dem Vakuumwiderstand empfohlen. Die Suspension kann dann mit nachfolgender Spülung mit Wasser über die Sonde appliziert werden [1, 7].

Zur Absicherung und zum Ausbau der verfügbaren Daten wurde auf Basis der Informationen die Sondierbarkeit für Capros® und Hydromorphon-HCl beta® weitergehend praktisch untersucht, wobei die beschriebene Methode als Grundlage für den Versuchsaufbau herangezogen wurde.

Die Applikation beider Präparate wurde jeweils unter Verwendung von HEC-Gel 4 % und 2 % mit der im Folgenden beschriebenen Spritzentechnik sowie mittels Trichter über gastrale Sonden Ch 10 und 8 geprüft. Die für die Testung verwendeten Sonden sind in Tab. 1 zusammengefasst. Es handelt sich bei den Testungen um Laborversuche (*ex vivo*).

#### Applikation mittels Spritzentechnik

Zunächst wurde die Retardkapsel geöffnet und der komplette Inhalt in einen Messbecher überführt. Der Spritzenkolben einer 10 ml-Spritze (ENFit™ bzw. Nutrisafe je nach verwendeter Sonde) wurde entfernt, die Auslassöffnung verschlossen und mit 5 ml Gel befüllt. Nach dem sogenannten Sandwich-Prinzip wurden zuerst die Pellets in die Spritze überführt und diese danach mit Gel auf ca. 10 ml aufgefüllt. Anschließend der Stempel wieder aufgesetzt, die Spritze entlüftet, auf die Sonde aufgeschraubt und der gesamte Spritzeninhalt in die Sonde entleert. Zur Spülung der Sonde wurden 10 ml Wasser verwendet.

#### Applikation mittels Trichter

Die Retardkapsel wurde geöffnet und der komplette Inhalt in einen Messbecher überführt. Die Sonde wurde mithilfe einer Spritze mit 2 ml HEC-Gel vorgefüllt und anschließend ein Trichter aufgeschraubt. Der Kapselinhalt wurde in den Trichter überführt. Nach ca. 1 min wurde der Trichter entfernt, zunächst mit 10 ml HEC-Gel und anschließend mit 10 ml Wasser gespült.

Bei beiden Applikationstechniken wurde der Durchfluss der Pellets durch die Sonde beobachtet. Der Inhalt wurde in einem Becherglas aufgefangen und gemäß den Vorgaben vernichtet. Die Sonde wurde nach der Applikation auf mögliche zurückbleibende Pellets untersucht.

## Ergebnisse

### Qualitätskontrolle der Dosisteilung durch HPLC-Analytik

**Abb. 3:**
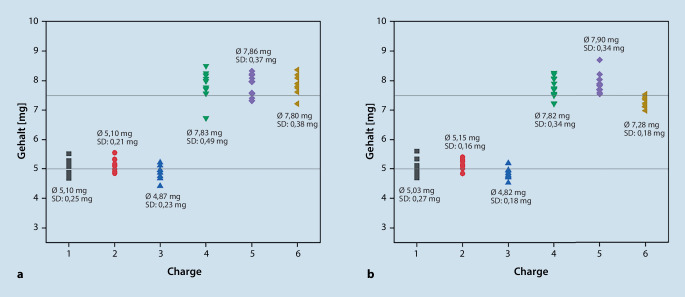
Gehalt von 10 einzeldosierten Morphinkapseln (entspricht einer Charge), dargestellt als Punkte im Diagramm. Sechs Morphinsulfat-Chargen sind im September 2021 hergestellt worden (**a**), 6 weitere im Dezember 2021 (**b**). Zusätzlich zum durchschnittlichen Gehalt pro Charge (Ø) ist die Standardabweichung (SD) angegeben (**a**, **b**). Zur Überprüfung, ob der Arzneistoffgehalt in den entsprechend abgewogenen Pellets den Sollwerten (5 mg oder 7,5 mg) entspricht, ist der Arzneistoff Morphinsulfat mittels HPLC quantifiziert worden (**a**, **b**). Das Europäische Arzneibuch erlaubt Abweichungen zwischen 85 und 115 % des Durchschnittsgehalts, wobei ein Einzelgehalt von 10 Einheiten außerhalb der Grenzen (85–115 %) des Durchschnittsgehalts liegen darf

Unter Anwendung der oben beschriebenen validierten HPLC-Methode sind insgesamt 12 Morphinsulfat-Chargen mit jeweils 10 Proben zur Überprüfung des Arzneistoffgehalts analysiert worden. In Abb. 3 sind die ermittelten Gehälter aller Chargen als Punkte im Diagramm dargestellt. Zusätzlich angegeben sind der durchschnittliche Gehalt pro Charge und die Standardabweichung.

Die größten Abweichungen aller Morphinsulfat-Chargen liegen bei −11,6 % (88,4 %) und 15,9 % (116 %). Nach EuAB entspricht die Prüfung auf Gleichförmigkeit des Gehalts einzeldosierter Arzneiformen bei Kapseln, wenn nur ein Einzelgehalt von 10 Einheiten außerhalb von 85 bis 115 % des Durchschnittsgehalts und keiner außerhalb von 75 bis 125 % liegt. Demnach sind die Anforderungen nach EuAB erfüllt [3].

**Abb. 4:**
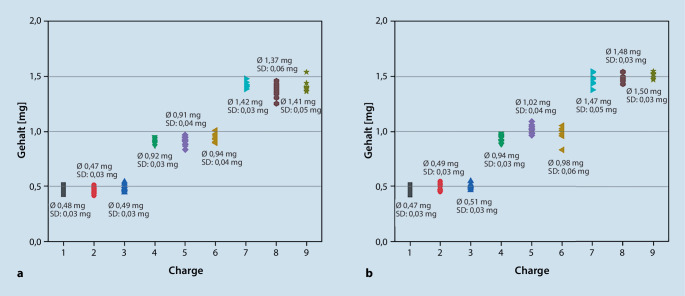
Gehalt von 10 einzeldosierten Hydromorphonkapseln (entspricht einer Charge), dargestellt als Punkt im Diagramm. Neun Hydromorphon-HCl-Chargen sind im August 2021 hergestellt worden (**a**), 9 weitere im Dezember 2021 (**b**). Zusätzlich zum durchschnittlichen Gehalt pro Charge (Ø) ist die Standardabweichung (SD) angegeben (**a**, **b**). Zur Überprüfung, ob der Arzneistoffgehalt in den entsprechend abgewogenen Pellets den Sollwerten (0,5 mg, 1 mg oder 1,5 mg) entspricht, ist der Arzneistoff Hydromorphon-HCl mittels HPLC quantifiziert worden (**a**, **b**)

In Abb. 4 sind die ermittelten Gehälter nach Analyse der insgesamt 18 Hydromorphon-Chargen ebenfalls als Punkte dargestellt. Auch hier sind der durchschnittliche Gehalt und die Standardabweichungen im Punktdiagramm notiert.

Die größten Abweichungen aller Hydromorphon-Chargen liegen bei −17,8 % (82,2 %) und 8,95 % (109 %). Auch hier liegt kein Einzelgehalt außerhalb der Grenze, sodass auch hier die Anforderungen des EuAB erfüllt sind.

### Prüfung auf Sondierbarkeit

Die Applikation mittels HEC-Gel 4 % erwies sich bei Sonden mit Ch 8 und 10 aufgrund der hohen Viskosität als zu schwergängig. Der erforderliche Druck war sehr hoch. Der Kraftaufwand wurde daher für die Praxis als zu groß eingeschätzt. Bei Verwendung von HEC-Gel 2 % war hingegen kein Widerstand vorhanden.

Mittels Spritzentechnik konnten die Capros®-Pellets bei allen getesteten Sonden (Ch 8 und 10) problemlos appliziert werden. Es blieben keine Pellets in der Sonde zurück. Die Hydromorphonpellets konnten ebenfalls rückstandsfrei durch alle Sonden mit Ch 10 gegeben werden sowie durch die Flocare®-Sonden mit Ch 8. Bei der Applikation durch Nutrisafe2®-Sonden mit Ch 8 blieben hingegen einzelne Pellets an der Auslassöffnung zurück. Durch den geringeren Durchmesser der Auslassöffnungen sowie die seitliche und nichtterminale Orientierung im Vergleich zu PEG-Sonden konnten nicht alle Hydromorphonpellets die Sonde passieren, sodass bei der Applikation ein Okklusionsrisiko besteht.

Mittels der Applikation über den Trichter konnte Capros® unter Beachtung der Gesamtmenge der auf einmal in den Trichter überführten Pellets über Sonden Ch 10 appliziert werden. Bei Applikation des gesamten Kapselinhalts (entsprechen 10 mg Morphinsulfat) kam es zu einer Okklusion, welche mit mehrmaligem Spülen mittels HEC-Gel 2 % aufgelöst werden konnte. Nichtsdestotrotz stellt dies ein Risiko dar. Bei Applikation von nur 5 mg (halber Kapselinhalt) trat hingegen keine Okklusion auf. Falls eine Gesamtmenge von über 5 mg benötigt wird, ist daher eine Aufteilung der Dosis auf 2 Gaben, zwischen denen mit HEC-Gel 2 % gespült wird, dringend zu empfehlen. Eine Applikation über Sonden mit Ch 8 war nicht möglich.

Hydromorphon-HCl war nicht über Trichter applizierbar, da die Pellets zu groß sind, um den Trichter zu passieren.

Eine Applikation der retardierten Opioidpräparate Capros® und Hydromorphon-HCl beta® über die untersuchten Sonden mit Ch 10 und zum Teil auch über Sonden mit Ch 8 ist unter Berücksichtigung der beschriebenen Vorgehensweise im Laborversuch möglich. Eine Zusammenfassung der Ergebnisse stellt Tab. 2 dar.

**Tab. 2:** Übersicht der Sondierbarkeit von Pellets aus Capros®- und Hydromorphon-HCl-beta®-Retardkapseln

Sonde	Applikation von Capros® mittels Spritzentechnik	Applikation von Hydromorphon-HCl beta® mittels Spritzentechnik	Applikation von Capros® mittels Trichter	Applikation von Hydromorphon-HCl beta® mittels Trichter
Flocare® Pursoft Tube (Nutricia) Ch 8	✔	✔	✘	✘
Flocare® Nutrisoft Tube (Nutricia) Ch 8	✔	✔	✘	✘
Flocare® Pursoft Tube (Nutricia) Ch 10	✔	✔	✔ (Aufteilung der Dosis ab > 5 mg)	✘
Flocare® Nutrisoft Tube (Nutricia) Ch 10	✔	✔	✔ (Aufteilung der Dosis ab > 5 mg)	✘
Nutrisafe2® PUR (Vygon) Ch 8	✔	✘	✘	✘
Nutrisafe2® PUR (Vygon) Ch 10	✔	✔	✘	✘
Freka FKJ (Fresenius)	✔	✔	✘	✘

## Diskussion

Ziel dieser Untersuchung war es, Patienten, Ärzten und Pharmazeuten bei fehlenden Daten aus der Pharmaindustrie einen Handlungskorridor für die Therapie mit retardierten Opioidpräparaten als Alternative zu MST®-Retardgranulat zur Verfügung zu stellen, der eine adäquate Versorgung dieser vulnerablen Patientengruppe von schwer kranken Kindern oder auf eine Ernährungssonde angewiesenen Erwachsenen ermöglicht. Mit unserem vorgeschlagenen Prozedere ist es mit den aktuell auf dem Markt verfügbaren und getesteten Präparaten (Capros®-10 mg-Hartkapseln retardiert und Hydromorphon-HCl-beta-2 mg-Retardkapseln) möglich, Patienten, die eine retardierte Opioideinzeldosis von Morphin < 10 mg oder Hydromorphon < 2 mg benötigen, sicher zu versorgen. Es sollte berücksichtigt werden, ob die Rotation oder der primäre Einsatz eines transdermal wirksamen Präparats (Fentanyl, Buprenorphin) für die Erkrankungs- und Versorgungssituation des Patienten eine Alternative sein könnte. Für sehr junge Kinder bedeutet die Abschaffung des MST®-Retardgranulats, dass nur noch schnell wirksame Präparate eingesetzt werden können, wodurch die Therapiequalität vermindert sein kann.

**Abb. 5:**
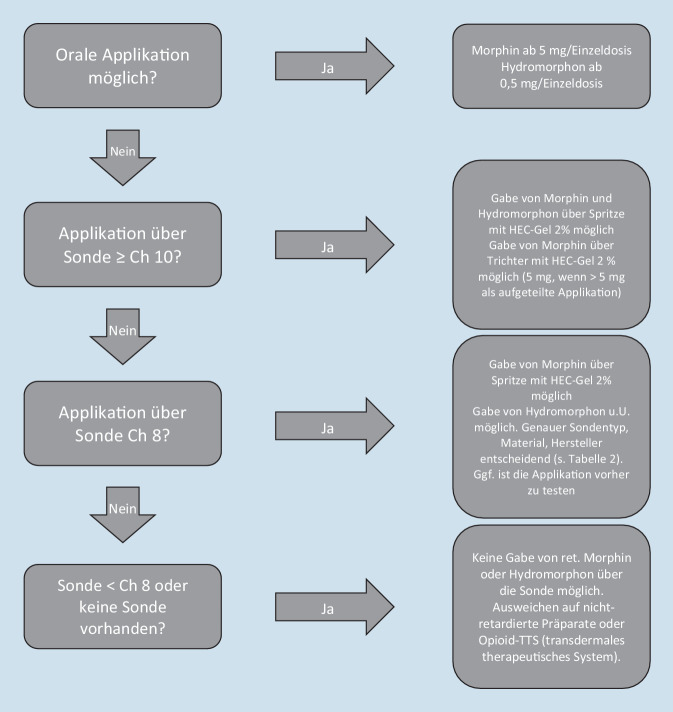
Entscheidungshilfe zum Einsatz von retardierten Opioiden

Unsere *Ex-vivo*-Untersuchung liefert eine erste Grundlage, sodass nach entsprechender Aufklärung der Eltern für den „off label use“ ein Schema vorliegt, welches in der Praxis umgesetzt werden kann (Abb. 5). Wir weisen explizit darauf hin, dass es sich hierbei lediglich um eine Empfehlung auf Basis von Laborversuchen handelt. Die Entscheidung und Verantwortung über die Therapie („off label use“) liegt daher weiterhin in den Händen des behandelnden Arztes.

Um die Therapiesicherheit bei hier nicht untersuchten Sondentypen bzw. Herstellern der gleichen Charrière-Größe nicht zu gefährden, ist im Einzelfall eine Testung vor Therapiestart zu empfehlen, da sich die Innendurchmesser und Auslassöffnungen der verschiedenen Sonden trotz identischer Charrière-Größen unterscheiden können.

Weitere Faktoren *in vivo* wurden nicht berücksichtigt. Wir empfehlen grundsätzlich eine Beachtung der notwendigen Flüssigkeitsgaben für die Medikamentenapplikation insbesondere bei neonatologischen Patienten.

Für die Umsetzung des in dieser Arbeit vorgestellten Vorgehens ist eine intensive Kooperation zwischen den entsprechenden Apothekern, den klinischen Anwendern und den betroffenen Familien, als Experten für die Versorgung ihrer Kinder, notwendig. Sinnvoll erscheint es uns, in den entsprechenden Foren der Spezialisten die Erfahrungen mit diesem gewählten Vorgehen zu sammeln und auszuwerten.

## Fazit für die Praxis

Die Untersuchung zeigt, dass das eingesetzte Herstellungsverfahren für die individuell dosierten Abfüllungen eine gute Dosiergenauigkeit liefert. Das Verfahren kann damit als validiert und sicher angesehen werden und bietet somit eine Möglichkeit, in der Pädiatrie retardierte stark wirksame Opioide auch über gastrointestinale Sondenapplikation einzusetzen.
